# Traumatismes iatrogènes de l'artère fémorale secondaire à la pose du cathéter d'hémodialyse à propos de 4 cas

**DOI:** 10.11604/pamj.2015.20.329.5979

**Published:** 2015-04-07

**Authors:** Rachid Zaghloul, Hamza Naouli, Jiber Hamid, Abdelatif Bouarhroum

**Affiliations:** 1 Service de Chirurgie Vasculaire, CHU Hassan II, Fès, Maroc

**Keywords:** Traumatisme iatrogène, artère fémorale, cathéter, hémodialyse, iatrogenic trauma, femoral artery, catheter, hemodialysis

## Abstract

Le recours aux cathéters veineux centraux (CVC) est incontournable en hémodialyse. Comparé aux autres abords vasculaires, il est pourtant associé à une morbidité et une mortalité plus élevées. Selon une étude menée par El Minshawy et coll, l'atteinte de l'artère fémorale représente 5% de ces complications. Nous en rapportons 4 cas ayant été pris en charge au sein du service de chirurgie vasculaire. L'objectif de ce travail est d’élucider les aspects cliniques, anatomopathologiques et les modalités thérapeutiques.

## Introduction

Le recours au cathéter d'hémodialyse est inévitable en attendant la création ou la maturation d'un abord vasculaire permanent, des données récentes ont montré qu'entre 15 à 30% des patients en insuffisance rénale chronique terminale débuteront leurs séances de dialyse par cathéter veineux central (CVC) [1]. Selon une étude menée par El Minshawy et coll, l'atteinte de l'artère fémorale représente 5% de ces complications [2]. Quand l'hémodialyse est indiquée en urgence, la veine fémorale est le site le plus simple et le plus facile pour l'insertion du cathéter d'hémodialyse [1, 3]. Cependant il n'est pas dénué de complications pouvant être dramatiques.

## Patient et observation

### Observation 1

**Figure 1:**
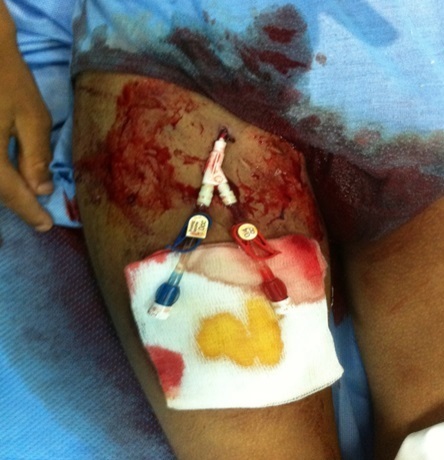
Image en préopératoire montrant le KT avec saignement actif

**Figure 2:**
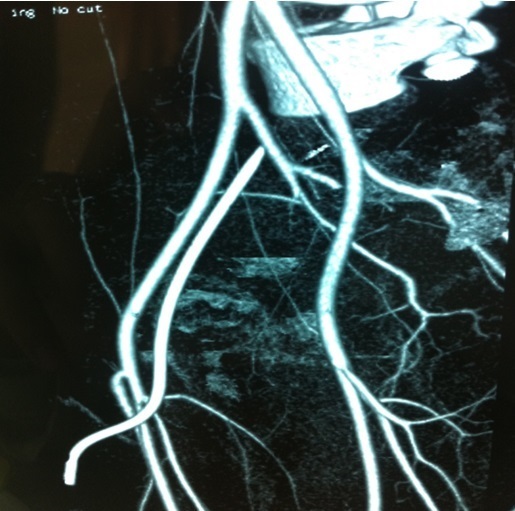
Angioscanner montrant la traversée transfixiante du KT au niveau de l'artère fémorale

Patient âgé de 18 ans, Suivi pour IRCT au stade d'hémodialyse, admis aux urgences pour prise en charge d'une ischémie aigue du membre inférieur droit avec un saignement actif suite à une pose d'un cathéter fémoral d'hémodialyse, l'angiographie a montré la traversée transfixiante du cathéter au niveau de l'artère fémorale superficielle avant de rejoindre la veine fémorale (Figure 1, Figure 2). Le patient a été opéré en urgence, on a réalisé l'ablation du KT d'hémodialyse et le rétablissement de la continuité artérielle. La difficulté de la pose du KT d'hémodialyse chez ce patient et l'urgence de cet abords vasculaire ont nécessité la mise en place chirurgicale du KT en controlatéral.

### Observation 2

**Figure 3:**
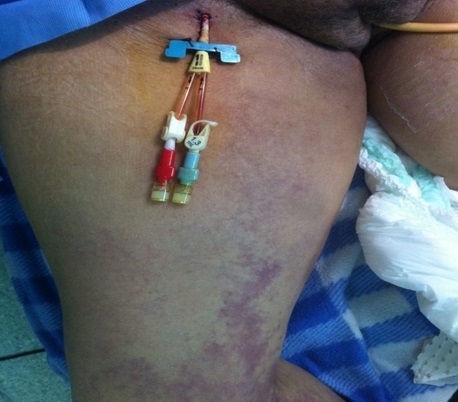
Image montrant le KT d'hémodialyse et la cyanose par endroit remontant jusqu’à la cuisse

**Figure 4:**
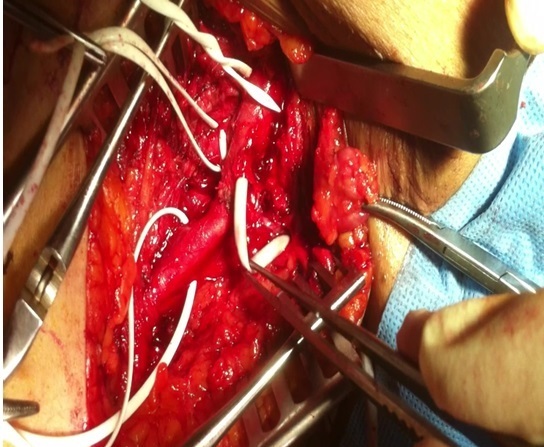
Le retrait chirurgical du KT qui siège à l'intérieur de l'artère fémorale commune

Femme de 60 ans, ayant comme antécédents une IRCT en hémodialyse et un diabète type 2, admise aux urgences pour prise en charge d'une ischémie aigue avancée du membre inférieur droit. L'examen clinique a objectivé un membre pâle, froid associé à une cyanose par endroit et un déficit sensitivomoteur (Figure 3). Les pouls poplités et distaux étaient abolis tandis que le pouls fémoral était présent. Vue la mise en jeu du pronostic fonctionnel du membre, la patiente fut acheminé au bloc opératoire sans investigations radiologiques préalables. L'exploration chirurgicale avait montré le passage exclusif du cathéter au niveau de l'artère fémorale commune (Figure 4). On a réalisé le retrait du cathéter avec une embolectomie à la sonde de Fogarty du trépied fémoral droit et la suture de la brèche. L’évolution a été marquée par la consommation du membre, l'amputation transfémorale était malheureusement la seule alternative thérapeutique.

### Observation 3

Patiente de 79 ans. Suivie pour une hypertension artérielle, un diabète type 2 et une IRCT en hémodialyse depuis 9 ans, consulte pour une dyspnée lentement progressive, des douleurs du pied droit rebelles au traitement antalgique et un début de nécrose du gros orteil. L'examen clinique objective une nécrose sèche du gros orteil avec abolition des pouls poplités et distaux et perception d'un thrill au niveau du Scarpa. L'exploration angiographique a objectivé la présence d'une FAV entre le pédicule fémoral. Le traitement a consisté en l'ablation du cathéter avec l'exclusion de la fistule, suture des deux brèches artérielle et veineuse suivies par une amputation de l'hallux droit. L’évolution a été marquée par l'amélioration de la dyspnée, la disparition de la douleur et une bonne cicatrisation des troubles trophiques.

### Observation 4

**Figure 5:**
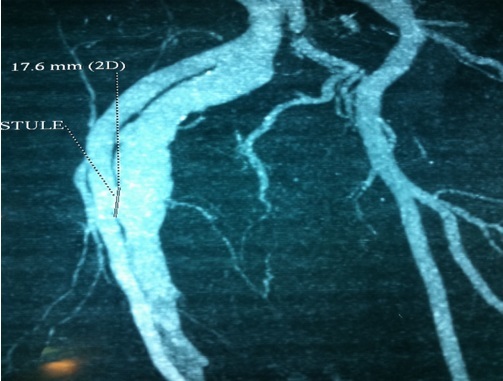
FAV du pédicule fémorale droit après pose de KT d'hémodialyse

**Figure 6:**
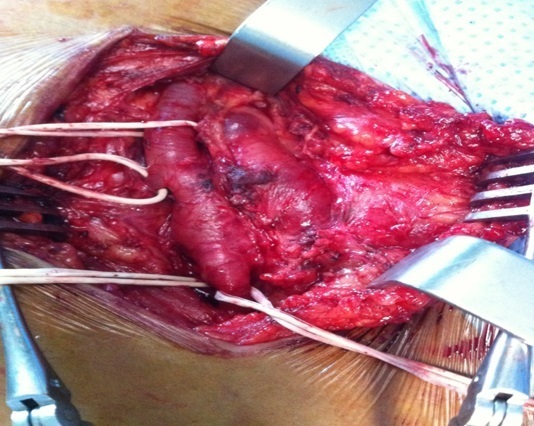
Contrôle chirurgical de la FAV

Patient âgé de 49 ans ayant comme ATCD une IRCT sur néphropathie hypertensive en HD, qui se plaine d'une dyspnée d'aggravation progressive et Perception d'un bruit anormal au niveau de l'aine. L'examen clinique a objectivé la présence d'un thrill au niveau du scarpa droit avec diminution des pouls. Les investigations radiologiques ont montré la présence d'une FAV fémoro-fémoral commune droite (Figure 5). Le patient a bénéficié de l'exclusion de la FAV avec reconstruction du trépied fémoral par l'interposition d'une prothèse en dacron (Figure 6). L’évolution a été marquée par la disparition du thrill, récupération de tous les pouls du membre inferieur droit, et l'amélioration de la dyspnée.

## Discussion

C'est une série, certes limitée en nombre, mais riche en variétés cliniques et anatomopathologiques. Le recours au cathéter d'hémodialyse est inévitable en attendant la création ou la maturation d'un abord vasculaire permanant, selon des données récentes entre 15 à 30% des patients en insuffisance rénale chronique terminale débuteront leurs séances de dialyse par cathéter veineux central (CVC) [2], (en France: 50%, aux USA: 70%). L'insertion d'un CVC au niveau du site fémoral se fait habituellement par ponction percutanée à l'aveugle aidée par la connaissance de l'anatomie habituelle des vaisseaux, l'existence de repères cutanés ou osseux et, le cas échéant, la palpation du poul de l'artère de voisinage [4]. Selon une étude menée par David et all, plus de 15% des malades ayant bénéficié d'un cathétérisme veineux central présentent au moins une complication, Ces complications sont d'ordre infectieux dans 5 à 26%, thrombotiques dans 2 à 26%, ou mécaniques dans 5 à 19% des cas [5]. Le traumatisme iatrogène de l'artère fémorale représente 5% des complications de la pose d'un CVC fémorale selon EL Minshawy et coll [1]. L'incidence de ces complications mécaniques varie en fonction de multiples facteurs: L'inexpérience, de relation variable mais constante avec la fréquence de ces complications, en effet L'expérience de l'opérateur joue, certes, un rôle dans la survenue de ces complications mécaniques. Ainsi, Sznajder démontre que lorsqu'un opérateur effectue plus de 50 poses de cathéter veineux central, les complications mécaniques diminuent d'environ 50% [6]. Autrement, Eisen et collaborateurs démontrent que lorsqu'un opérateur dépasse deux tentatives de pose, le risque de survenue d'une complication mécanique passe de 17% à 54% [7]. D'autres facteurs peuvent aussi intervenir à savoir un indice de masse corporelle supérieur à 30 ou inférieur à 20, des cathétérisations antérieures, une déshydratation sévère ou une hypovolémie, une coagulopathie, Un cathéter de grande taille, et une chirurgie ou une radiothérapie antérieure au niveau de la région anatomique intéressée [8]. Giswold et al., ont montré que l'atteinte de l'artère fémorale au cours des procédures percutanées (Tableau 1) était prédominante avec un taux atteignant les 80% [9].

**Tableau 1:** Répartition du traumatisme artérielle au cours de 62 procédures percutanées

Artère	Nombre (%)
Fémorale	50 (80,6%)
Brachiale	5 (8,1%)
Sous-clavière	3 (4,8%)
Radiale	2 (3,3%)
Axillaire	1 (1,6%)
Carotide	1 (1,6%)

La connaissance des repères anatomiques sont donc primordiaux pour éviter les complications surtout d'ordre mécanique. Mais, malheureusement, la technique classique du repérage anatomique n'est pas dénuée de complications, lesquelles contribuent à une augmentation des durées et des coûts d'hospitalisation et majorent le risque de survenue de complications secondaires [10]. Pour limiter l'incidence de ces complications immédiates, des équipes anglo-saxonnes ont démontré l'utilité de l'usage de l’échographie bidimensionnelle [11]. Le bénéfice de ce guidage ultrasonographique pour l'abord veineux central est aujourd'hui bien établi chez l'adulte [12]. Ainsi, s'appuyant sur une méta-analyse récente [12], le National Institute for Clinical Excellence (NICE) britannique a diffusé les recommandations suivantes [13]: 1) Le guidage de la ponction par l’échographie bidimensionnelle est recommandé lors de la mise en place des cathéters veineux centraux par voie jugulaire interne chez l'adulte et chez l'enfant; 2) dans les situations d'urgence, la possibilité de recourir aux ultrasons devrait être considérée; 3) une formation appropriée des professionnels susceptibles de placer des cathéters veineux centraux sous écho-guidage est recommandée; 4) l'utilisation du doppler sonore comme mode de guidage n'est pas recommandée, ce qui rejoint les recommandations EBPG 2007 Guideline 10.2. [14], qui insiste sur l'importance de l'utilisation de l’écho-guidage lors de l'insertion des KT provisoires et de longue durée par voie percutanée (Tableau 2) [15]. La stratégie thérapeutique, après un cathéter aberrant, inclue une simple compression locale, une réparation endovasculaire ou une chirurgie ouverte qui reste la technique de choix la plus sûre mais souvent considérée comme lourde pour ce genre de patients dont la majorité est porteuse de tares multiples. L'alternative thérapeutique reste, sans doute la prévention. Nous retenons comme points essentiels l'utilisation de l’écho-guidage, éviter de ponctionner très proche du pouls fémoral, l'usage des aiguilles fines pour ponctionner, et Si ponction de l'artère fémoral, éviter l'héparine pendant les 24 heures.

**Tableau 2:** Écho-guidage Vs repères anatomiques voie fémorale

	Repères N = 55	Echo N = 55
Succès (%)	80	98,2
Succès 1^ère^ tentative	54,5	85,5
Nombre tentatives	1,52	1,16
complications	18,2	5,5

## Conclusion

Le traumatisme iatrogène de l'artère fémorale secondaire à la pose du cathéter d'hémodialyse est une complication mécanique redoutable et peut être évité par l'utilisation de l’écho-guidage au cours de la procédure. Toute suspicion d'un cathéter intra-artériel doit mener à la réalisation d'investigations radiologiques toutefois le délai le permet.
